# Utilization of Liposuction for Delayed Morel-Lavallée Lesion: A Case Report and Review

**DOI:** 10.1155/2017/8120587

**Published:** 2017-09-11

**Authors:** Preston Gardner, Diana Flis, Kongkrit Chaiyasate

**Affiliations:** ^1^Department of Plastic & Reconstructive Surgery, Beaumont Hospital-Farmington Hills, 28050 Grand River Ave, Farmington Hills, MI 48336, USA; ^2^Department of Plastic & Reconstructive Surgery, Detroit Medical Center, 4160 John R., Ste 400, Detroit, MI 48201, USA; ^3^Department of Plastic & Reconstructive Surgery, Beaumont Hospital-Royal Oak, 3555 West 13 Mile Rd., Ste N120, Royal Oak, MI 48073, USA

## Abstract

Morel-Lavallée lesions are irregularly occurring and often overlooked results of traumatic injuries, resulting in potential long-term encapsulation of fluid between soft-tissue layers. The objective in this review was to discuss the delayed presentation of a Morel-Lavallée lesion and operative utility of liposuction in the patient's treatment and review literature with particular focus on diagnosis and therapeutic interventions. The reviewed case demonstrates the presentation and successful therapy of a young female presenting with a MLL and contour deformity.

## 1. Introduction

Clinically described in 1863 by French surgeon Victor Auguste François Morel-Lavallée, the Morel-Lavallée lesion (MLL) is an infrequently diagnosed injury. MLL most commonly results from blunt force trauma causing shearing of the deep soft-tissue layers typically between adipose and fascia. Shearing trauma may subsequently lead to the creation of a pocket between soft-tissue layers, leading to encapsulation and/or contour deformity. Providers often refer to MLL as a posttraumatic pseudocyst or closed soft-tissue degloving injury [1, 2]. 

## 2. Case Report

**Figure 1:**
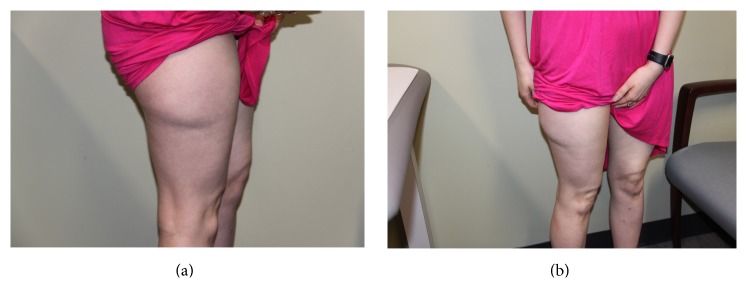
(a) Preop lateral. (b) Preop oblique.

A 31-year-old female was referred to the outpatient Plastic Surgery Clinic due to an abnormal contour deformity of the right thigh. She had previously sustained injury as a pedestrian two years before referral, after being struck by a motor vehicle, and while down her right thigh, she was ran over by a second motor vehicle. Her chief complaint was a right thigh mass/deformity, which she noted to be chronically painful. On physical examination, she was noted to have a soft palpable enlarged area on the lateral right thigh measuring 15 × 15 cm; however, there were no local or systemic signs of infectious process (Figure 1).

**Figure 2:**
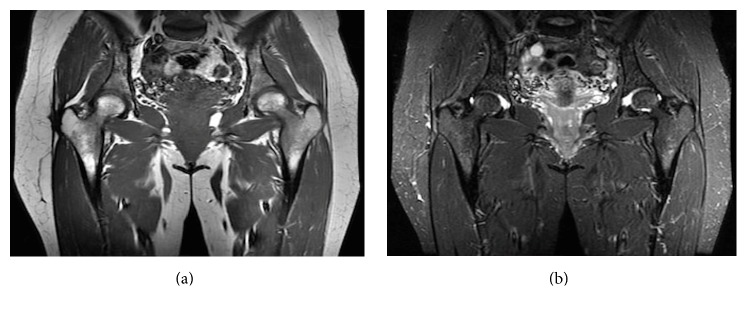
(a) Coronal T1 MRI. (b) Coronal T2 MRI.

Magnetic Resonance Imaging (MRI) revealed a 1.7 × 0.7 × 5.5 cm fluid-filled structure along the iliotibial tract with tethering of the adjacent skin (Figure 2).

The patient underwent suction-assisted lipectomy with utilization of tumescent fluid of the right thigh. The soft-tissue area was circumferentially marked preoperatively. General anesthesia was given and the patient was placed in the left lateral decubitus position. Three-millimeter incisions were made at the anterior border of the deformity and at the posterior border. Tumescent fluid was infused into the soft tissues and then three-millimeter cannulas were utilized for liposuction until satisfactory intraoperative contour. Incisions were closed with simple interrupted 5-0 polypropylene sutures and then wrapped with an ACE wrap for compression. The patient was discharged home the same day.

A follow-up appointment was conducted in the office one week postoperatively. There was a moderate amount of ecchymosis along the lateral right thigh; however, the previously noted contour abnormality was markedly improved. The patient had mild residual postsurgical pain but was satisfied with the early results.

**Figure 3:**
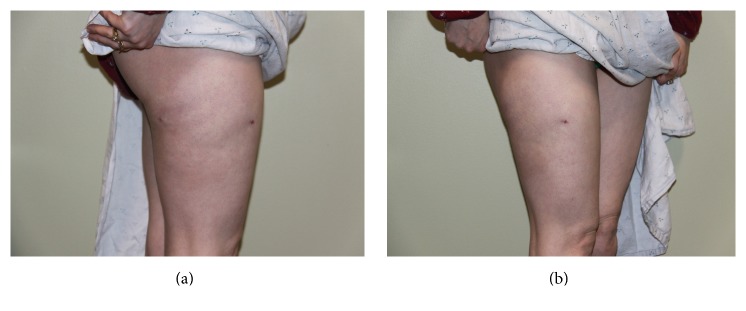
(a) Postop lateral. (b) Postop oblique.

Seven weeks postoperatively, another follow-up appointment occurred. The previously noted ecchymosis had completely resolved, the contour deformity remained considerably improved, and she was exceptionally pleased with her outcome (Figure 3). Patient was again seen for follow-up at five months postoperatively. She remains very satisfied with the aesthetic appearance of the contour, with her only complaint being residual neuropathy, unchanged from preoperative symptoms.

## 3. Discussion

### 3.1. Pathophysiology

As previously noted, MLL most frequently occurs following blunt force trauma. The disturbance causes a shearing effect of soft-tissue layers adjacent to fascia. Shearing may lead to dead space between intact underlying fascia and the more superficial soft tissues. In addition, the space allows the accumulation of blood, necrotic fat, lymphatic fluid, and other inflammatory mediators. These types of lesions/accumulations have been described following high-energy trauma, such as motor vehicle incidents, assaults, sport-related injuries [1, 2], and surgical procedures, such as abdominoplasty, with [3, 4] and without [5] liposuction.

Patient presentations of MLL have been demonstrated to occur as early as the first few days or even as late as 13 years after initial suspected traumatic insult [6, 7]. In a review of the literature, Vanhegan et al. demonstrated the most common locations of involvement, being the hip/greater trochanter at 36% and thigh at 24% [8]. Other case series have similarly demonstrated higher predilection for lower extremity involvement with 60% [9] up to 81% [10]. If MLL remains untreated, a prolonged inflammatory process may ensue instigating formation of a pseudocyst leaving potential for infection and necrosis [1].

### 3.2. Diagnosis

While a thorough history and physical exam may lead to suspected diagnosis, often times adjunct radiographic imaging being utilized. Multiple medical settings use plain film X-ray radiography and have found it useful. Plain film X-ray radiography may be beneficial in the immediate posttraumatic setting to evaluate for adjacent fractures, such as femur or pelvis, but however, remains nonspecific for MLL [1]. In the setting of MLL, plain films may demonstrate mass effect of soft tissue without calcification or fat layer infiltration [11].

Ultrasonography has also been used in MLL evaluations. Neal et al. demonstrated that 21 of 21 patients had lesions located between the deep layer of fat and fascia but, however, were of variable appearance: 71% hypoechoic and 29% anechoic, 62% heterogeneous and 38% homogenous, and 60% fusiform, 25% flat, and 15% lobular. While ultrasonography may assist in the initial MLL evaluation, it has not been shown to be a definitive diagnostic radiological source [12].

During initial trauma evaluations within the emergency department, the importance of computed tomography can be seen because it is frequently and readily available. In the early posttraumatic period, computed tomography may reveal MLL as a hypodense fluid collection within the deep soft-tissue planes with fluid layering [13]. In chronic MLL, there has been shown to be a more defined surrounding capsule of the fluid-filled layering [14].

Magnetic resonance imaging (MRI) is argued as the diagnostic imaging of choice, particularly when dealing with a chronic lesion [15]. On MRI, MLL have a variety of radiographic findings including the following: sharply distinct borders, including a hypointense peripheral rim which may be thin, thick, or absent; a laminar, oval, linear, or round morphology; fusion of margins to adjacent fascia; internal septations; and internal fluid layering of variable signal intensity. Mellado and Bencardino developed a six-type classification system based on MRI findings and complexity but, however, does not address direction of treatment [16].

### 3.3. Treatment

Multiple therapeutic interventions have been discussed in the literature ranging from percutaneous drainage to open excision. Due to the rarity of MLL, most therapies are discussed in case report format and few retrospective reviews, without significant level 1 or level 2 evidence. The majority of case reports entail open excisional evacuation and debridement as the definitive therapy, with or without prior attempts at conservative compression and/or percutaneous drainage and with or without discussion of intraoperatively placed drainage tubes [17–20].

In a review of 22 patients, Carlson et al. discussed their treatment modality for patients with MLL with open wounds or within a surgical field. After open excision and debridement, they obliterate the cavity dead space with suture every 4-5 cm^2^ (with absorbable suture if the fat layer was thick or with nonabsorbable suture over dental bolsters if there was little to no remaining fat layer) and postoperative drain maintenance until less than 30 mL output per 12 hours. Their cohort developed no infections or reaccumulation; however, there were two patients with superficial flap necrosis successfully treated with local wound therapy [21]. Other case reports have noted a similar technique with the goal of decreasing the dead space via open incisions and debridement and then subsequent “quilting sutures” of the capsule all with stated no recurrence, good cosmetic result, and/or no further symptoms of their single patients [8, 20, 22].

Many argue that a minimally invasive approach may be the best modality. Large open incision and debridement procedures may tend to injure the vascular supply to an already traumatically compromised soft-tissue area, thus predisposing to poor wound healing and/or soft-tissue necrosis [23]. This is mostly in regard to lesions treated in an acute setting, as chronic lesions may not have such a tenuous blood supply, with adequate time to undergo neovascularization. With a minimally invasive approach in mind, some have formulated utilization of sclerodesis as therapy for MLL. Luria et al. described their cohort of 4 patients, with average of three-month lesion duration, all diagnosed with physical exam and computed tomography imaging and all with recurrent fluid collection following simple percutaneous aspiration. Percutaneously, the cavity was drained, sent for culture, and then instilled with a talc solution for five minutes, and a drain was left in place postoperatively. With an average 27-month follow-up, there was no long-term fluid reaccumulation; however, one patient required a second talc therapy, who was* Staphylococcus aureus* positive on fluid culture [24]. Bansal et al. utilized doxycycline for sclerodesis in 16 patients with follow-up ultrasound postoperatively. Eleven patients had total fluid resolution at four weeks after sclerodesis, four patients with resolution at eight weeks, and one patient required a second sclerodesis procedure at 12 weeks, allegedly attributable to noncompliance with compression wraps. At an average of 50.44-month follow-up, there was no demonstration of instances of recurrence, infection, or skin necrosis [10]. Yet another described sclerodesis approach was by Penaud et al. in a five-patient cohort diagnosed with MRI and treated with limited 2 cm incision and drainage of the cavity, irrigation with hydrogen peroxide, then instillation of pure ethanol, and drain placement. At six months postoperatively, all remained asymptomatic, and, on follow-up MRI, four patients had complete resolution of fluid collection and one patient had residual fluid noted 3-fold smaller than preoperatively [25].

Nickerson et al. described management guidelines setup at Mayo Clinic based on a 79-patient retrospective review. They noted a recurrence rate of 56% in percutaneously drained MLL versus 19% in conservatively treated MLL and 15% after operative debridement. More importantly, they noted that, in lesions that recurred in percutaneously drained patients, greater than 50 mL had been aspirated in 83% of them versus only 33% in patients without recurrence. They consequently recommended that if a MLL was percutaneously drained of at least 50 mL operative therapy was warranted [7]. Another proposed algorithmic approach recommended by Dawre et al. involves the following: ascertaining the duration of the lesion's presence, association with open versus closed fracture, and presence or absence of infection. The algorithms involve a progressive approach with initiation of conservative measures, such as compression, to percutaneous drainage with or without sclerotherapy and eventually open drainage/debridement if unresolving [26].

In addition to all the previously discussed treatment modalities for MLL, suction-assisted lipectomy has also found a role, however sparsely appearing in the literature. Liu et al. discussed a single case of MRI confirmed MLL on the lateral upper arm. They treated with a 5 mm trocar inserted into the lesion, irrigation, visualization with endoscope, then liposuction following soft-tissue tumescence with no clinical signs of recurrence at three-month follow-up [27]. Hudson also discussed his series of seven patients with a closed degloving injury treated with liposuction. His clinical description of the clinical findings resembles that of MLL; however, no radiographic information was given to confirm diagnosis in the article. His explicated therapy involved liposuction of the associated area with five of seven with satisfactory improvement, one of which required a second liposuction procedure and two of the seven subsequently required open incision for contour correction [6].

## 4. Conclusion

As an infrequently occurring soft-tissue injury, there remains a paucity of high-level evidence on the subject of MLL. Most literature revolved around case reports/series and retrospective reviews, with little headway for prospective trials due to the uncommonly presenting nature of the lesion. In our patient, liposuction proved itself as a successful method to reestablish improved contour, likely through release of scar and contracture, and, however, unlikely to impact the chronic pseudocyst itself. However, given that the patient's chief complaint was the misshapen appearance of her thigh and high degree of postoperative satisfaction, other similarly presenting lesions may likewise be amenable to a comparable approach.
